# Journalistic Notes

**Published:** 1891-09

**Authors:** 


### Journalistic Notes.

A MAGAZINE WRITTEN BY WOMEN.—As was expected, the greatest interest has been exhibited in the new story by Amélie Rives which was begun in the August number of the *Cosmopolitan*. The second part appears in the September number and the story will close in the following issue.

The September issue of the *Cosmopolitan* Magazine is a "woman's number" so far as the authorship of its articles is concerned, but the general interest of the periodical is sustained by the variety and timeliness of the topics treated. The opening article, on Edouard Detaille, is by Lady Dilke, and is profusely and beautifully illustrated with reproductions of the famous artist's most noteworthy paintings. A Forgotten City, by Eleanor Lewis, is a romantic description of the ruins of Soluntum, the Sicilian Pompeii, embellished with photographs. Malmaison in the Market, by Mary Bacon Ford, describes the waning fortunes of the house celebrated for the residence there of the ill-fated Empress Josephine. Julia Hayes Percy describes the Ladies' New York Club in an entertaining article, to which Harry Fenn has contributed illustrations. Elizabeth Bisland writes of Tattersall's, the great London horse market, and the family who have given it name and fame. Molly Elliott Seawell contributes The Romance of

Count Königsmark, the titled adventurer for whom the wife of George I. of England spent thirty years in prison; and the Countess Ella Norraikow writes of Woman's Share in Russian Nihilism, her article being illustrated with portraits of many fair conspirators. There are, besides, papers on the Evolution of the Society Journal, by Mrs. Roger A. Pryor; Society Women as Authors, by Anna Vernon Dorsey; a pretty story, Il Mandolinista, by Daisy O'Brien, and verses by Katherine Grosjean, Mrs. Charles B. Foote, and Susan Hartley Swett, all the important articles being liberally illustrated.

---

THE *New England Medical Monthly* has issued its September number in souvenir form, to commemorate its tenth anniversary. It is printed on heavy plate paper, and contains an autotype frontispiece of the Editor-in-Chief and his associate editors. Besides, scattered through the journal are full-page plates of a large number of the distinguished physicians of the United States and foreign countries. This souvenir number is a monument to the ability, generosity, and indefatigability of Dr. William C. Wile, who conducts this interesting and valuable journal.

---

THE *Climatologist* has made its appearance. It is a monthly journal of medicine, devoted to the relation of climate, mineral springs, diet, preventive medicine, race, occupation, life insurance, and sanitary science to disease, edited by John M. Keating, M. D., J. P. Crozer Griffith, M. D., and Charles F. Gardiner, M. D., with a number of distinguished associate editors. It is published by W. B. Saunders, 913 Walnut street, Philadelphia, Pa.

---

*Anales del Departaments Nacional de Higiene* is the name of a new journal just published in Buenos Aires. It is devoted to the study of hygienic questions, legal medicine, bacteriology, climatology, meteorology, etc. Judging from its first number, it is bound to be a success, and in keeping with the high standard enjoyed by the medical journals of the Argentine capital.

